# Prompt Engineering and ChatGPT: Delivering Quality Information to Dementia Caregivers

**DOI:** 10.1093/geroni/igaf122.2254

**Published:** 2025-12-31

**Authors:** Stephanie Bennett, Alex Fedorov, Rasheeta Chandler, Kenneth Hepburn, Fayron Epps

**Affiliations:** Emory University, Atlanta, Georgia, United States; Emory University, Atlanta, Georgia, United States; Emory University, Atlanta, Georgia, United States; Emory University, Atlanta, Georgia, United States; UT Health San Antonio, San Antonio, Texas, United States

## Abstract

Dementia caregivers rely on the internet for access to quick information. Increasingly, they are turning to prompt-based large language models (PBLLMs) like ChatGPT to improve outcomes for persons living with dementia (PLWD). However, researchers highlight significant limitations in ChatGPT’s responses to health-related questions, including contextual understanding, emotional awareness, readability, and transparency. To overcome these shortcomings, prompt engineering (PE) —the practice of crafting specific, detailed prompts to guide a PBLLM—has emerged as promising strategy. In this novel study, we used web content analysis to evaluate 15 responses from ChatGPT. Specifically, we assessed the effectiveness of using PE with ChatGPT based on aforementioned limitations by comparing responses to five general caregiving questions and two iterations (10) of PE statements. Questions focused on five topics: websites, specialists, behavioral issues, tests, and medications. We maintained the topics but revised each question using Cumming’s PE method, which required details about reference websites, ChatGPT’s role, caregivers’ goals, context, and tasks. The first set of PE-statements (5) included context about the PLWD. The second set (5) incorporated the additional context of race. Study findings showed that responses to general questions promoted engagement by including follow-up questions. Although the PE responses lacked these follow-ups, they provided contextually relevant information to help caregivers achieve their goals and fostered emotional awareness. These responses demonstrated improved readability and included links to credible sources. Our findings suggest that PE methods can enhance ChatGPT’s limitations, improving responses for caregivers of PLWD and paving the way for future methodological research in AI-assisted communication.

